# Exploring the Molecular Mechanism of Sepal Formation in the Decorative Flowers of *Hydrangea macrophylla* ′Endless Summer′ Based on the ABCDE Model

**DOI:** 10.3390/ijms232214112

**Published:** 2022-11-15

**Authors:** Qi Wang, Tong Lyu, Yingmin Lyu

**Affiliations:** 1 Beijing Key Laboratory of Ornamental Plants Germplasm Innovation & Molecular Breeding, China National Engineering Research Center for Floriculture, College of Landscape Architecture, Beijing Forestry University, Beijing 100083, China; 2 Beijing Flower Engineering Technology Research Center, Plant Institute, China National Botanical Garden North Park, Beijing 100093, China

**Keywords:** flower development, *Hydrangea macrophylla*, floral organ development gene, molecular biology

## Abstract

With its large inflorescences and colorful flowers, *Hydrangea macrophylla* has been one of the most popular ornamental plants in recent years. However, the formation mechanism of its major ornamental part, the decorative floret sepals, is still not clear. In this study, we compared the transcriptome data of *H. macrophylla* ‘Endless Summer’ from the nutritional stage (BS1) to the blooming stage (BS5) and annotated them into the Kyoto Encyclopedia of Genes and Genomes (KEGG) and Gene Ontology (GO) databases. The 347 identified differentially expressed genes (DEGs) associated with flower development were subjected to a trend analysis and a protein–protein interaction analysis. The combined analysis of the two yielded 60 DEGs, including four MADS-box transcription factors (*HmSVP-1*, *HmSOC1*, *HmAP1-2*, and *HmAGL24-3*) and genes with strong connectivity (*HmLFY* and *HmUFO*). In addition, 17 transcription factors related to the ABCDE model were screened, and key candidate genes related to the development of decorative floret sepals in *H. macrophylla* were identified by phylogenetic and expression pattern analysis, including *HmAP1-1*, *HmAP1-2*, *HmAP1-3*, *HmAP2-3*, *HmAP2-4,* and *HmAP2-5*. On this basis, a gene regulatory network model of decorative sepal development was also postulated. Our results provide a theoretical basis for the study of the formation mechanism of decorative floret sepals and suggest a new direction for the molecular breeding of *H. macrophylla*.

## 1. Introduction

*Hydrangea macrophylla* is a perennial deciduous shrub of *Hydrangea* in Hydrangeaceae, which is popular for its full flower shape and colorful flowers. It is not only a common potted ornamental flowering shrub, but it also can tolerate semishady environments and is an important part of the understory in many areas, with high ornamental and ecological values. The cymes of *H. macrophylla* include sterile flowers and fertile flowers. Sterile flowers, also known as decorative florets, are decorative mainly because of their large, showy sepals that help attract the attention of pollinators, which is also their main ornamental trait. Fertile flowers, on the other hand, also known as nondecorative florets, have small and inconspicuous sepals and are mainly responsible for seed production [1]. Since decorative florets greatly enhance the ornamental value of *H. macrophylla*, many breeders have made this a breeding and production goal, hoping to increase its number through various methods. The *H. macrophylla* variety ‘Endless Summer’ has remontancy characteristics and can be grown in relatively cold northern regions, making it one of the most common hydrangea varieties available at present. Moreover, its nondecorative flowers have an unstable withering phenomenon during flower development (personal observation), which is also a good material for studying the development of decorative flowers.

As an important reproductive organ of angiosperms and the main ornamental part of ornamental plants, flowers have always been the focus of research by botanists due to their diverse appearance and rich variation types [2,3]. Additionally, in the study of plant flower development, the morphogenesis process of flowering organs involves a complex multigene molecular genetic regulatory network, which is a hot field of this research [4]. The floral organ characterization genes were first identified in *Arabidopsis thaliana* and *Antirrhinum majus* [5]. An analysis of floral homozygous mutants in both led to the formation of the genetic ABC model in the early 1990s [6,7]. In terms of floral anatomy, complete flowers of dicotyledons generally consist of four whorls of concentric structures, in order from the outside in: the 1st whorl of sepals, the 2nd whorl of petals, the 3rd whorl of stamens, and the 4th whorl of carpels [5]. The ABC model predicts that the combined action of three types of homologous heterozygous floral organ homology genes determines the identity of the four floral organ types [7]. Subsequently, researchers have identified class D genes, which are closely related to class C genes and have similar expression patterns and partially overlapping functions [8,9], as well as class E genes that are necessary for the development and formation of four rounds of floral organs and have partial functional redundancy [3,9,10]. Therefore, the ABCDE model of flower development was formally formed.

Among the five classes of ABCDE genes, all of them share a commonly conserved region and belong to the MIKC^C^-type MADS-box genes in the MADS-box gene family, except *APETALA2 (AP2)*, a member of the class A gene [4]. The MADS-box gene family is an important family of transcription factors that are widely distributed in nature and whose members commonly play a role in growth and developmental regulation and signaling in eukaryotes [11]. The main role of these genes first identified and studied in plants was to regulate the development of floral organs and flowering time. The members of the MADS-box family all have a conserved domain composed of 56–58 amino acids in their protein structure, called the MADS-box [12]. MADS-box genes can be classified into two major types, Type I and Type II, based on phylogenetic relationships, gene structure, and protein structure. Type I MADS-box genes usually contain 1–2 exons, and the encoded protein contains only one highly conserved MADS domain [13], while Type II MADS-box genes generally include six introns and seven exons, which are composed of four domains of M (MADS), I (Intervening), K (Keratin-like), and C (C-terminal), also known as the MIKC-type gene [12,14,15]. In plants, Type II MADS-box genes can be subdivided into MIKC^C^-type genes and MIKC*-type genes [14], and Becker et al. [16], by phylogenetic analysis of MIKC^C^-type MADS-box genes in angiosperms, classified them into 12 major subfamilies, which include five classes of floral homeotic genes that control organ homology during floral organ development in the ABCDE model. Subsequent experiments, such as gel blocking and yeast two-hybrid, showed that these five classes of genes formed homo- or heterodimer through MIKC^C^-type MADS structural domain proteins, which followed by ternary or quaternary protein complexes to determine the formation of different floral organs [9]. Thus, the floral quartet model of floral organ development was proposed. This model not only validates and refines the ABCDE model but also explains the interactions between proteins related to floral organ development at the protein level, providing a favorable basis for the study of the molecular mechanism of floral organ development.

Sepal morphology is the most significant difference in appearance between decorative and nondecorative flowers of *H. macrophylla*. During sepal formation, all sepals of nondecorative flowers were initiated successively within a comparatively short period, while a single sepal of decorative flowers was initiated on the abaxial side first, and other sepals were initiated after the single sepal had developed to a certain extent [17]. In addition, differences in the expression of meristem characteristic genes, such as *LEAFY (LFY)*, *APETALA 1 (AP1),* and *TERMINAL FLOWER 1 (TFL1)*, may also lead to the formation of decorative and nondecorative florets [17]. In monocots, such as Tulipa spp. and Agapanthus praecox, in addition to whorls 2 and 3, class B genes are also expressed in whorl 1 and are involved in regulating tepal morphogenesis [18,19]. In Ranunculaceae plants, there are also class B genes expressed in the petaloid sepals [20]. Consequently, some studies speculate that the formation of decorative sepals in H. macrophylla may be jointly regulated by class A and class B genes [21,22]. Furthermore, to resolve controversies over the ill-defined function of class A genes, there was also a study that proposed the (A) B (C) model, where (A) = A + E and (C) = C + D [23]. This model both regains the simplicity of the ABC model and generalizes and simplifies the floral quartet model. Even so, there are still few studies related to the genes of floral organ development in *H. macrophylla*, and the formation mechanism of its decorative florets has still not been elucidated.

In this study, differential genes at five flowering stages were investigated using transcriptome sequencing with *H. macrophylla* as the plant material. We focused on analyzing the main biological processes associated with flower development and then screened the genes related to the formation of decorative floret sepals to reveal the intrinsic molecular mechanism of decorative floret formation and provide a theoretical basis for the breeding of *H. macrophylla* with more decorative flowers.

## 2. Results

### 2.1. Phenotypic Observation

**Figure 1:**
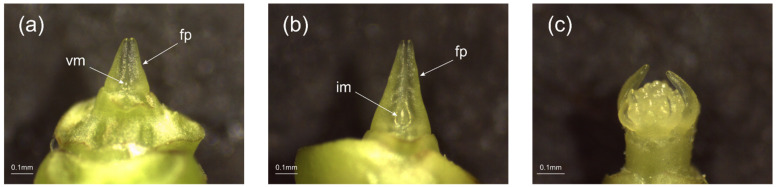

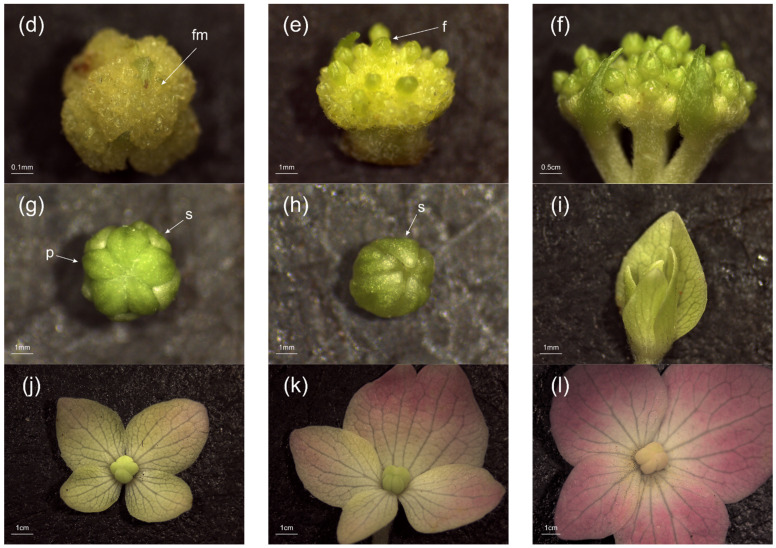
Flower development stages of *Hydrangea macrophylla* ‘Endless Summer’ observed by stereomicroscope. (**a**) Vegetative bud. (**b**) Inflorescence meristem dome formation begins. (**c**) Spherical shape formed by numerous inflorescence meristem domes. (**d**) Floral primordium differentiation stage. (**e**) Floral organ differentiation stage. (**f**) Secondary branching of spherical inflorescence. (**g**) Nondecorative floret on secondary inflorescence branching. (**h**) Decorative floret on secondary inflorescence branching. (**i**) Decorative floret with elongated sepals. (**j**) Decorative floret sepals unfold. (**k**) Decorative floret sepals start to turn color. (**l**) Decorative floret sepals completely changed color. vm = vegetative meristem; im = inflorescence meristem; fm = floral meristem; fp = foliar primordia; f = floret; s = sepal; p = petal.

The morphological characteristics of the sampled flower buds and inflorescences were observed to determine their differentiation status, and we selected the developmental stages with distinct differentiation characteristics for comparison. As shown in Figure 1, the development of decorative flowers of *H. macrophylla* started from the vegetative bud (Figure 1a) and gradually formed an inflorescence meristem dome (Figure 1b), which changed to the reproductive growth stage. Later, many inflorescence meristem domes were differentiated, forming a typical globular shape (Figure 1c). Subsequently, the differentiation of the floral primordia (Figure 1d) and the floral organs (Figure 1e) began. Figure 1f shows a part of the secondary inflorescence branching, when a clear spherical inflorescence formation could already be observed macroscopically. Figure 1g,h correspond to a nondecorative floret and a decorative floret in the inflorescence of Figure 1f, respectively. At this point, the sepals of the nondecorative floret were obviously shorter than the petals, and both could be observed and clearly distinguished at the same time, while the sepals of the decorative floret wrapped around the entire floret and the petals were located inside the sepals. Subsequently, the sepals of the decorative floret slowly elongated, changed color, and unfolded (Figure 1i–l), reaching the full flowering stage and completing the development of the floral organs.

### 2.2. Quality Analysis of Transcriptome Sequencing

**Figure 2:**
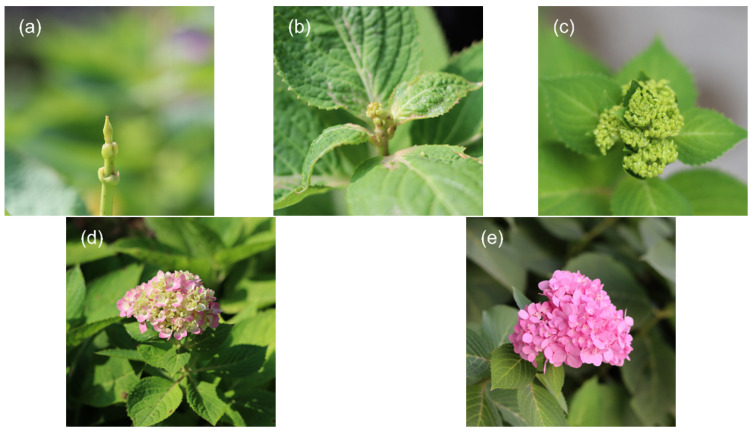
Flower phenotypes at different developmental stages. (**a**) BS1: vegetative bud, corresponding to Figure 1a. (**b**) BS2: flower bud, corresponding to Figure 1d. (**c**) BS3: formation of inflorescence, corresponding to Figure 1f. (**d**) BS4: initial flowering stage, corresponding to Figure 1k. (**e**) BS5: full blooming stage, corresponding to Figure 1l.

Five cDNA libraries were constructed by comparative transcriptome sequencing of floral organs of ‘Endless Summer’ at different developmental stages. Of these, BS1 and BS2 were mixed samples of vegetative buds and flower buds, respectively, and BS3 to BS5 were the mixes of decorative flowers collected during that growth period (Figure 2). The results show (Table 1) that the raw reads of each sequencing library were above 7 G, and the high-quality clean reads obtained after filtering were not less than 6 G. The number of valid bases of all tested samples was more than 94%; the GC content was approximately 45%, and Q30 ≥ 88.66%. This indicates that the bases are of high quality and the transcriptome sequencing is of good quality, meeting the requirements of subsequent assembly and data analysis.

### 2.3. Functional Annotation and Expression Analysis of Transcriptome Unigenes

#### 2.3.1. Analysis of KEGG Metabolic Pathway

**Figure 3:**
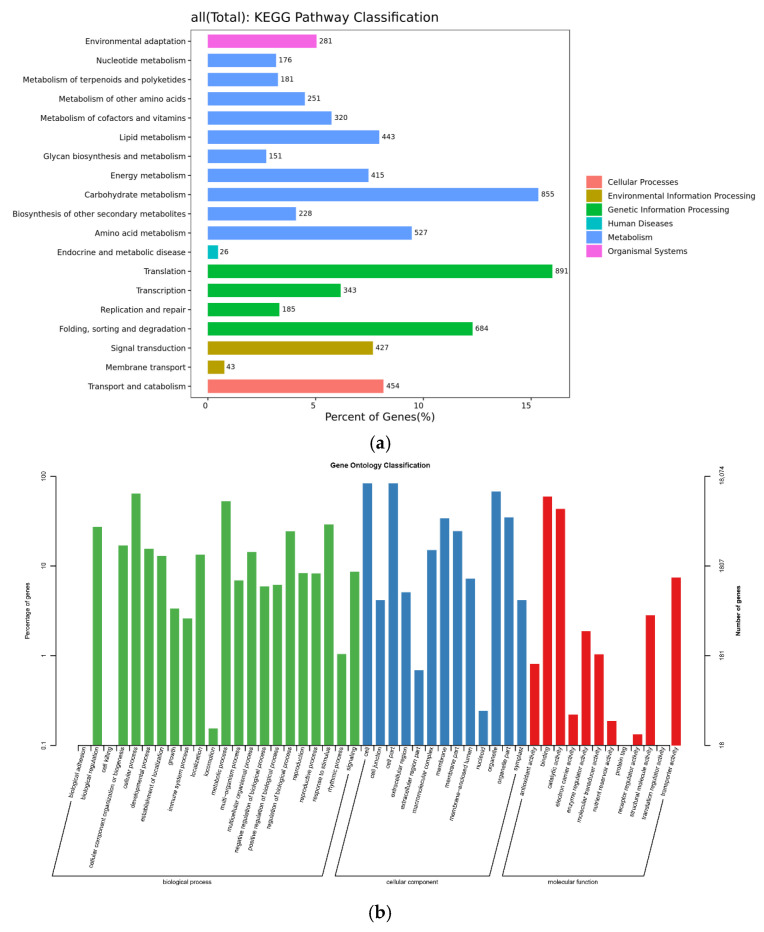
Annotation of unigenes in KEGG and GO datasets. (**a**) KEGGs. The horizontal axis represents the percent of genes annotated to the pathway; the vertical axis represents the Level 2 pathway name, and the number on the right of the bar represents the number of genes annotated to this Level 2 pathway. (**b**) GO. The horizontal axis represents the GO functional classification; the left vertical axis represents the percentage of genes annotated to that class, and the right vertical axis represents the number of genes annotated to that class.

To understand the specific function of unigenes obtained by transcriptome sequencing during the development of *H. macrophylla* and the metabolic pathways they involved, unigenes were mapped to the KEGG database (Figure 3a). The statistical results show that a total of 6881 unigenes were involved in 122 metabolic pathways, mapping to five types of branches of the KEGG pathway: metabolism, genetic information processing, environmental information processing, cellular processes, and organismal systems. The most active pathways in each branch include translation, carbohydrate metabolism, folding, sorting and degradation, amino acid metabolism, transport and catabolism, lipid metabolism, and signal transduction, which involve various aspects of plant life activities.

#### 2.3.2. GO Function Classification

The classification results of the unigenes obtained from this study after GO function annotation are shown in Figure 3b. A total of 18,121 unigenes from ‘Endless Summer’ were successfully annotated to 47 functional terms in three categories: molecular function (MF), cellular component (CC), and biological process (BP). Among them, the largest number of terms was annotated in the biological processes. According to a GO analysis, cellular process (11,589 entries) was the largest term of biological processes, followed by metabolic process (9527 entries) and response to stimulus (5244 entries). Among the cellular components, genes associated with cells (15,109 entries), cell parts (15,081 entries), and organelles (12,248 entries) predominated. Among the molecular functional categories, genes were mainly involved in binding (10,753 entries) and catalytic activity (7826 entries).

### 2.4. Screening and Enrichment Analysis of Differentially Expressed Genes

**Figure 4:**
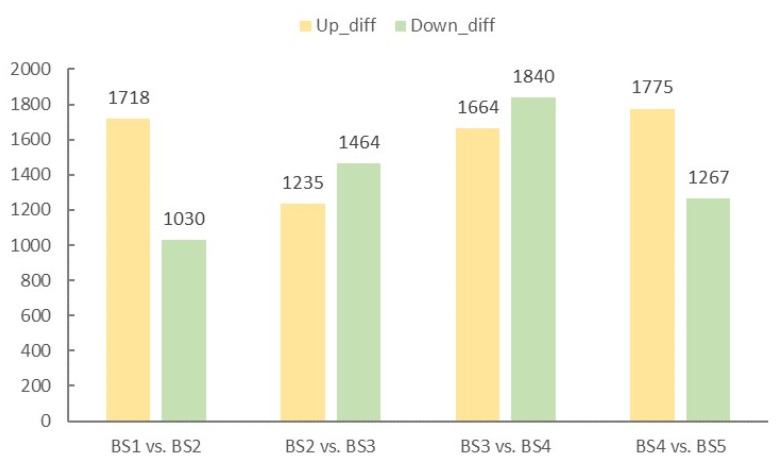
The number of differentially expressed genes among the four groups of samples. The horizontal axis represents the comparison group; the vertical axis represents the number of DEGs, and the yellow and green bars represent up- and downregulated genes, respectively.

Based on the results of the difference analysis, genes with *p*-value ≤ 0.05 and |log2Fold Change| ≥ 2 were screened as significantly different genes. From Figure 4, the number of upregulated differential genes was significantly higher than the number downregulated in BS1 vs. BS2 at the initial stage of floral organ development, which was probably related to the transition from vegetative growth to reproductive growth. The period from BS2 to BS4 belonged to the flower organ development period, and the DEGs related to flower development gradually increased, and the expression level increased. However, by late flower development, the expression of most DEGs related to floral organ development decreased, resulting in a higher number of differential genes being upregulated than downregulated in BS4 vs. BS5. With the continuous maturation of floral organs, the number of DEGs in the five developmental stages from leaf bud to decorative flower formation revealed a trend of increasing first and then decreasing, indicating that the DEGs screened in this study were related to flower organ development and could be used for a subsequent analysis of related gene identification. The up- and downregulated DEGs in the four comparison groups are shown in Supplementary Table S1.

**Figure 5:**
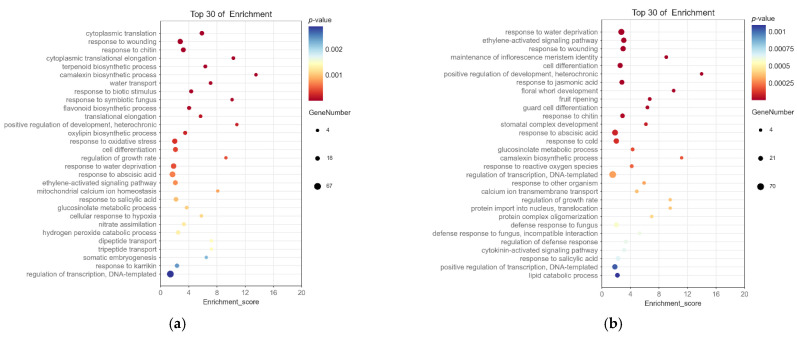

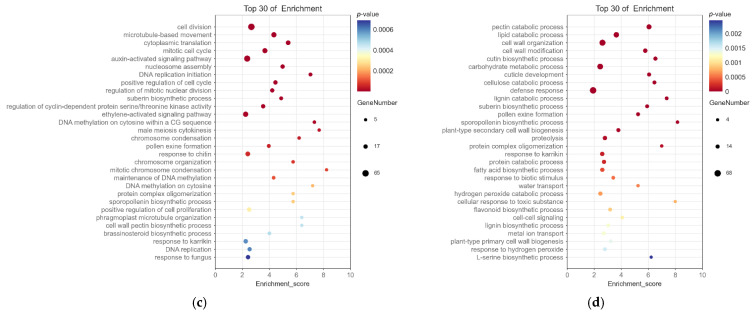
Bubble diagram of the top 30 biological process terms enriched in GO. The horizontal axis in the figure represents the enrichment score, and the vertical axis represents the terms name. The terms with larger bubbles contain a higher number of differential protein-coding genes. The bubble color changes from blue to red, and the smaller the enrichment *p*-value, the greater the significance. (**a**) BS1 vs. BS2. (**b**) BS2 vs. BS3. (**c**) BS3 vs. BS4. (**d**) BS4 vs. BS5.

The DEGs annotated with the GO function were enriched, and Figure 5 shows the 30 most significantly enriched GO terms for biological process (Supplementary Table S2). As shown in the figure, the main GO terms related to flower development enriched by each differentially expressed gene set were maintenance of inflorescence meristem identity (GO:0010077), floral whorl development (GO:0048438), and pollen exine formation (GO:0010584). Moreover, there were also 36 related terms, such as flower development (GO:0009908), regulation of flower development (GO:0009909), specification of floral organ identity (GO:0010093), and floral organ development (GO:0048437), which were enriched by DEGs.

### 2.5. Analysis of Differentially Expressed Genes Related to the Development of Decorative Flowers

Based on the DEGs in the process related to the formation of decorative flowers of *H. macrophylla*, combined with GO functional enrichment and other genes with significant differences or high expression, according to the functional annotation results and previous research results, this study screened out 347 DEGs related to flower development. Thereafter, through a trend analysis, PPI analysis, and phylogenetic analysis, we continued to narrow down the candidate genes and search for relevant key genes.

#### 2.5.1. Trend Analysis

**Figure 6:**
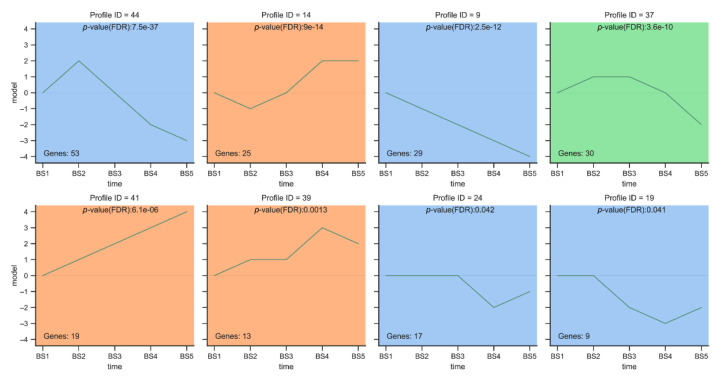
Trend analysis expression module of DEGs. The horizontal axis represents the five periods of flower development, and the vertical axis represents the changes in gene expression at different periods. Different colors indicate different expression patterns (*p* ≤ 0.05).

We performed a trend analysis on the expression patterns of the 347 DEGs associated with flower development screened above. The trend analysis divided the five developmental periods into 50 modules (Supplementary Figure S1), while the DEGs occupied a total of 37 modules, of which 8 were significantly enriched (Figure 6). A total of 11 MADS-box transcription factors and 78 other transcription factors from 23 transcription factor families were screened out from the eight significantly enriched modules (Supplementary Table S3). In modules 44, 9, 37, 19, and 24, the gene expression was generally upregulated followed by downregulation or downregulated, and all of them reached peak expression at the BS1 or BS2 stages. These genes may be the key genes involved in the formation of decorative sepals in *H. macrophylla*.

In Module 44 and Module 37, the DEGs showed an increasing first and then decreasing trend in the five periods of flower development (BS1–BS5). We screened a total of 20 other transcription factors from 11 transcription factor families in Module 44. Additionally, in Module 37, 1 MADS-box transcription factor (*HmAP1-2*) and 11 transcription factors from other families were screened. In Module 9, the DEGs tended to decrease continuously during five periods of flower development (BS1–BS5), and 1 MADS-box transcription factor (*HmJOIN*) and 17 other transcription factors from 12 transcription factor families were identified. In Module 19 and Module 24, the gene expression pattern of DEGs demonstrated a general trend of stable initially and then decreased during the five periods of flower development (BS1–BS5). Two MADS-box transcription factors (*HmSVP-1* and *HmSOC1*) and two other transcription factors were screened in Module 19. Additionally, one MADS-box transcription factor (*HmAGL24-3*) and five transcription factors from other families were identified in Module 24.

In summary, 5 MADS-box transcription factors and 55 other transcription factors were screened out from five significantly enriched trend modules 44, 9, 37, 19, and 24. These DEGs identified from the significantly enriched modules can be used for further analysis to narrow down the candidates for genes related to sepal development in the decorative florets of *H. macrophylla*.

#### 2.5.2. Protein–Protein Interaction (PPI) Analysis

Functional links between proteins can usually be inferred from genomic correlations between the genes encoding them, while a group of genes required for the same function tends to demonstrate similar species coverage.

**Figure 7:**
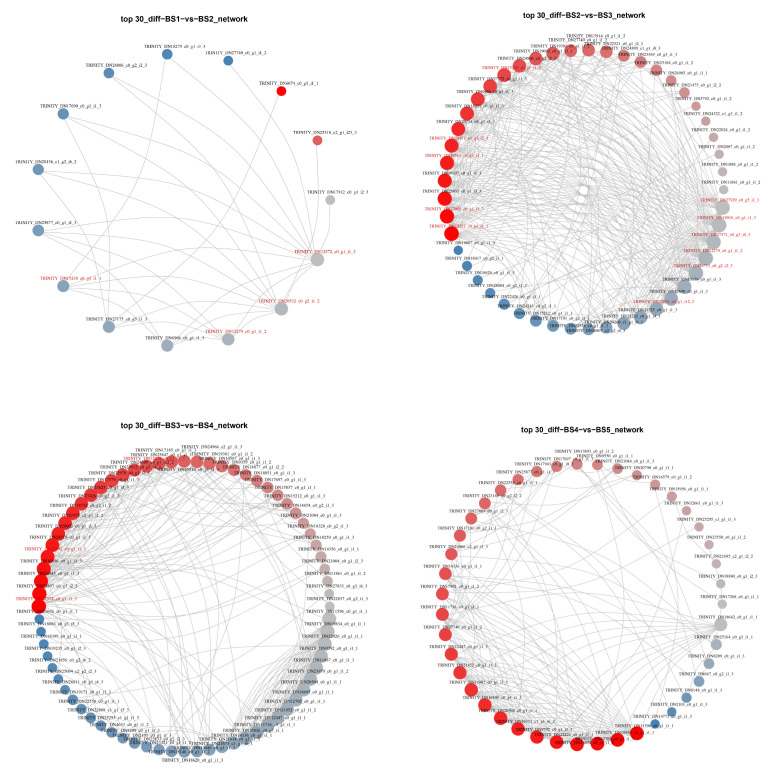
Protein–protein interaction network of differentially expressed genes. Red indicates differential genes with upregulated expression; blue indicates differential genes with downregulated expression, and the connecting lines between them indicate the presence of interaction between genes. The more genes that are associated, the larger the point of the gene.

We designed the PPI figure using 347 DEGs associated with flower development. According to the obtained protein–protein interactions (Figure 7), there were 15 DEGs with strong connectivity in the comparison between the BS1 and BS2 phases, of which five MADS-box transcription factors (*HmPI-1*, *HmMADS3*, *HmAP1-2*, *HmEJ2*, and *HmAGL19*) and six other transcription factors from five transcription factor families were screened. A total of 49 DEGs with strong connectivity were identified in the BS2 and BS3 phase comparisons, including 9 MADS-box transcription factors (*HmSVP-1*, *HmSOC1*, *HmSEP3-1*, *HmPI-1*, *HmAG-1*, *HmAGL15*, *HmSEP1*, *HmMADS3*, and *HmAP3-2*) and 22 other transcription factors from 16 transcription factors. In the comparison between the BS3 and BS4 phases, in total, 72 DEGs were more connected, of which we screened 24 other transcription factors from 16 transcription factor families. In the comparison between the BS4 and BS5 phases, there were 43 DEGs with strong connectivity, among which 1 MADS-box transcription factor (*HmAGL24-3*) and 13 other transcription factors from nine transcription factor families were identified. The PPI result files are shown in Supplementary Table S4.

According to the above PPI analysis, 179 DEGs were identified in the four sets of comparisons (Supplementary Table S5), and excluding 40 redundant genes, a total of 139 DEGs were obtained, including 13 MADS-box transcription factors and 51 other transcription factors. Combining them with the target modules in the trend analysis, we obtained a total of 60 DEGs, which included four MADS-box transcription factors (*HmSVP-1*, *HmSOC1*, *HmAP1-2*, and *HmAGL24-3*). Among the 60 DEGs screened, *HmLFY* (TRINITY_DN12572_c0_g1_i1_3) showed high connectivity in three groups. In BS1 vs. BS2, the four floral development-related genes, *HmLFY*, *HmAP1-2* (TRINITY_DN20532_c0_g2_i1_2), *HmUFO* (TRINITY_DN15238_c0_g1_i1_3), and *HmPI-1* (TRINITY_DN12279_c0_g1_i1_2), all had strong interactions with each other. In BS2 vs. BS3, *HmLFY*, *HmUFO*, *HmPI-1*, *HmAP3-2* (TRINITY_DN18016_c0_g1_i1_3), *HmSOC1* (TRINITY_DN11951_c0_g1_i2_1), *HmAP2-1* (TRINITY_DN27371_c0_g3_i4_3), *HmAG-1* (TRINITY_DN27929_c0_g5_i1_3), *HmSEP3-1* (TRINITY_DN24713_c0_g2_i2_3), *HmSVP-1* (TRINITY_DN10563_c0_g1_i1_1), *HmANT-2* (TRINITY_DN27092_c0_g1_i3_3), and *HmSEP1* (TRINITY_DN23823_c0_g1_i12_3) all showed strong correlations among the floral development genes. In BS3 vs. BS4, *HmLFY*, *HmUFO,* and *HmANT-2* interacted with one another strongly. Therefore, these DEGs can be used as a candidate gene set for screening the genes related to sepal formation in decorative florets of *H. macrophylla* for further analysis and research.

#### 2.5.3. Phylogenetic and Expression Pattern Analysis

**Figure 8:**
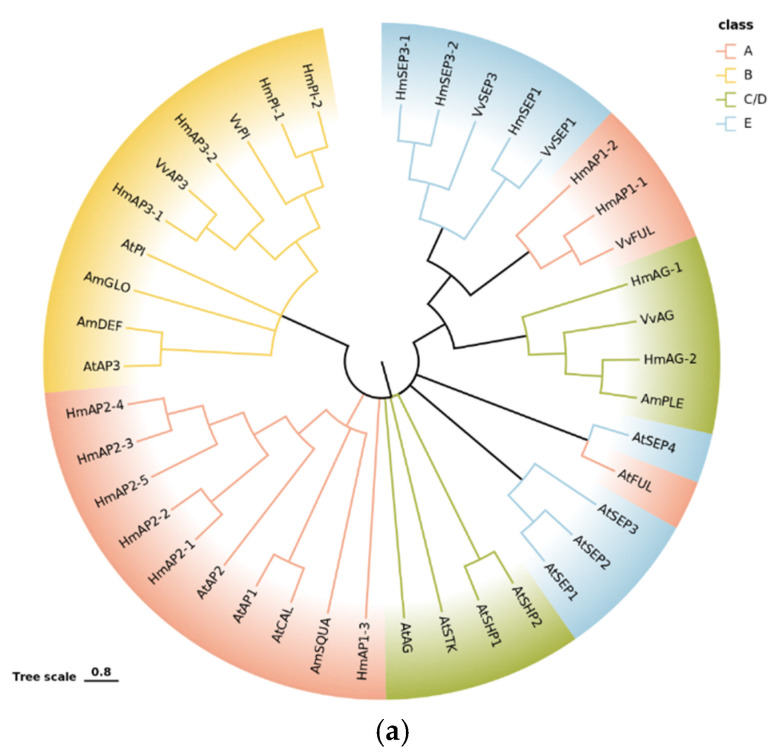

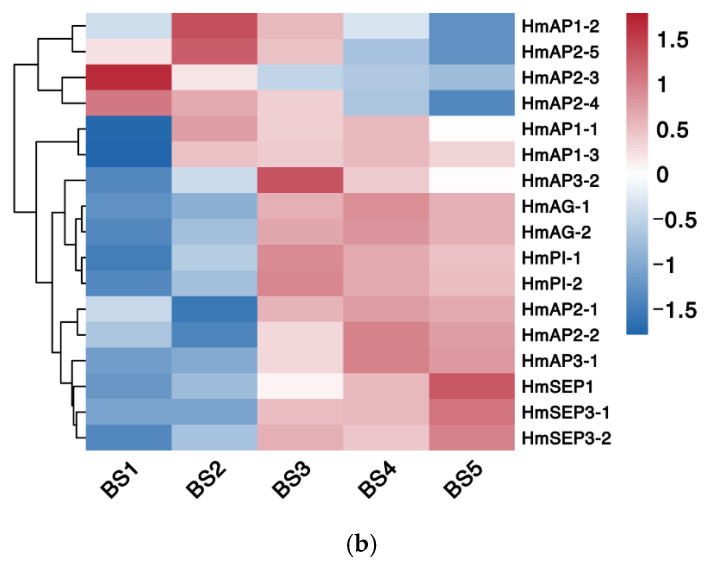
Phylogenetic and expression pattern analysis of genes associated with the ABCDE model. (**a**) Phylogenetic tree. (**b**) Cluster heatmap of DEGS related to the ABCDE model. The data preprocessing method takes the logarithm of the sequencing data with 2 as the base. A small square represents a gene, and its color represents the expression level of that gene. Red indicates high-expression genes, and blue indicates low-expression genes. The tree on the left side shows the results of clustering analysis of different genes from different samples.

Of the 347 DEGs mentioned above, 17 transcription factors associated with the ABCDE model were also screened (Supplementary Tables S6). A phylogenetic analysis of these 17 transcription factors with the ABCDE model-related genes of *Arabidopsis thaliana*, *Antirrhinum majus* and *Vitis vinifera* was performed, and the results are presented in Figure 8a. From the figure, the screened transcription factors were highly homologous with the ABCDE model-related genes of the representative plants, especially *Vitis vinifera*.

A hierarchical cluster analysis was performed on the screened transcription factors, and the genes with the same or similar expression profiles were clustered. The presented results are shown in Figure 8b. All DEGs were clustered into four groups, and the expression levels of the genes within the groups (i.e., the color in this figure, which represents the FPKM expression of the genes in the samples) were essentially similar. Class A genes were closely related to sepal development in the ABCDE model. A total of three *AP1* homologs and five *AP2* homologs were identified in *H. macrophylla*. Among them, *HmAP1-1* and *HmAP1-3* showed a similar expression pattern, both of which were elevated first and maintained at a high level after reaching a peak at the BS2 stage. The expression trends of *HmAP2-1* and *HmAP2-2* were similar and, in contrast to *HmAP1-1* and *HmAP1-3*, decreased first and then increased and maintained at a high level after reaching a minimum at the BS2 stage. Both *HmAP2-3* and *HmAP2-4* basically showed a continuous decreasing expression pattern. Meanwhile, *HmAP1-2* and *HmAP2-5* both showed a trend of increasing and then decreasing, and their expression peaked in the BS2 stage.

### 2.6. Real-Time Quantitative PCR (RT-qPCR) Verification of Differentially Expressed Genes

**Figure 9:**
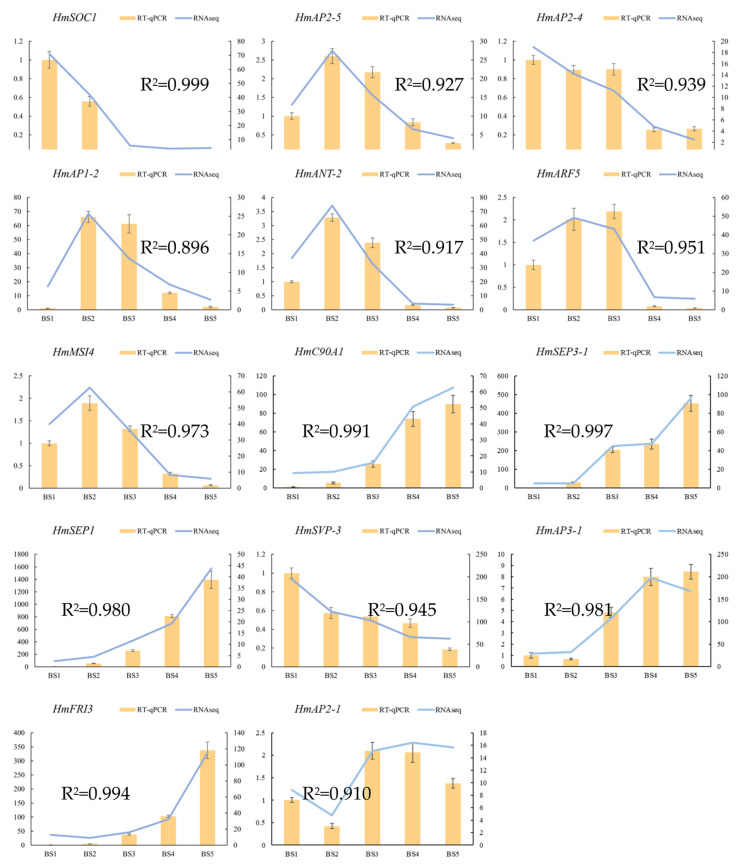
RT-qPCR validation of the expression patterns of 14 DEGs. The x-axis represents the five developmental stages; the left y-axis represents the relative expression levels of RT-qPCR results; the right y-axis represents the FPKM values from RNA-seq. Error bars show the standard error between three biological replicates (*n* = 3).

To verify the accuracy of the transcriptome sequencing results, this study selected 14 genes (*HmSOC1*, *HmAP2-5*, *HmAP2-4*, *HmAP1-2*, *HmANT-2*, *HmARF5*, *HmMSI4*, *HmC90A1*, *HmSEP3-1*, *HmSEP1*, *HmSVP-3*, *HmAP3-1*, *HmFRI3,* and *HmAP2-1*) that potentially related to flower development from the DEGs screened in the transcriptome data for RT-qPCR detection. The results show that the expression of the 14 genes by RT-qPCR at different flower developmental stages was basically consistent with the transcriptome sequencing results. Their relative expression levels were closely correlated with the FPKM values, and the Pearson’s correlation coefficients of both were above 0.896, which proved the high reliability of this sequencing (Figure 9). In addition to the 7 genes associated with the ABCDE model already mentioned above, RT-qPCR validation was also performed for the remaining 10 genes (Supplementary Figure S2). The results are in general consistent with transcriptome sequencing and can be used for subsequent studies.

## 3. Discussion

In this study, combined with morphological observation, a total of 17 ABCDE model-related transcription factors, including eight class A genes, four class B genes, two class C/D genes, and three class E genes, were identified by comparative transcriptome analysis of different developmental stages of *H. macrophylla*.

Following the origin of core dicotyledons, the evolution of features, such as a clear distinction between the petals and sepals in the perianth and the arrangement of floral organs, all have been associated with the duplication of class A and E genes [24,25]. *Arabidopsis thaliana* has two class A genes: *AP1* and *AP2*, of which only *AP1* encodes the MADS-box transcription factor. In addition to being expressed in the first sepal and second petal whorls, *AP1* also plays an important role in the formation of floral meristems, both as a characteristic gene for floral organs and for floral meristems [5,26]. In *H. macrophylla*, the *AP1* homolog produced three genes of this type, *HmAP1-1*, *HmAP1-2*, and *HmAP1-3*, through two small-scale gene duplications. Among them, *HmAP1-2* was highly expressed mainly in the BS2 stage, while *HmAP1-1* and *HmAP1-3* were expressed in both the BS2 to BS5 stages (Figure 8b). However, it was found that in the *Arabidopsis ap1* mutant, no ectopic expression of *AG* occurred, whereas in the *ap2* mutant, the expression of *AG* extended to whorls 1 and 2, demonstrating that there was no antagonistic relationship between *AP1* and *AG* and only the *AP2* gene had the function of inhibiting the *AG* gene [27]. It could be speculated that *HmAP1-2* may be mainly involved in the development of the sepals of *H. macrophylla*, while *HmAP1-1* and *HmAP1-3* may be involved in petal development in addition to sepal development, and their expression in the BS4 and BS5 stages did not affect the expression of class C/D genes in the same stage. Furthermore, within the AP1 subfamily of the MADS-box gene family, there are two *AP1* paralogous genes, *CAL (CAULIFLOWER)* and *FUL (FRUITFULL)*, and all three together form the *SQUA-like (SQUAMOSA)* gene [11,28]. *CAL* can positively regulate *AP1* expression, but all its functions are redundant with those of *AP1* [16,26,29]. In contrast, *FUL* has evolved with a function in valve identity specification [28]. Phylogenetic reconstructions suggest that the ancestral function of *SQUA-like* genes was to specify the identity of floral meristems, whereas the function to specify the identity of sepals and petals or fruit petals was derived later [30,31]. Kitamura et al. also analyzed three hydrangea *AP1* homologous genes in a study on morphological changes in anthurium flower organs induced by phytoplasma infection and speculated that the changes in their expression levels might contribute to bract formation in the pedicel [21]. However, no significant changes in class A genes were observed in that study, and it was also speculated that the class B gene, along with the class A gene, might jointly regulate the morphogenesis of decorative sepals in hydrangeas [21]. Additionally, the floral meristem determination gene *LFY (LEAFY)*/*FLO (FLORICAULA)* is also homologous to *AP1/SQUA*, with relatively high homology at both the DNA and protein levels, and *LFY* also activates *AP1* for expression throughout the floral meristem [3,32]. In the study by Nashima et al., they assumed that *LFY* was a causative gene of the double flower phenotype of ‘Sumidanohanabi’ [33]. In the decorative flowers of hydrangea, sepals showed petaloid characteristics, including pigmentation and organ enlargement. Additionally, the double flowers derived from *dsu* exhibited male sterility and reduced female fertility similar to the *Arabidopsis lfy* mutant [33]. The absence of petals and stamens in the *lfy* mutant flowers in *Arabidopsis* was traced back to a failure to activate the petal- and stamen-specific B-class genes *APETALA3* (*AP3*) and *PISTILLATA* (*PI*). To activate the gene expression of *AP3*, *UNUSUAL FLORAL ORGANS* (*UFO*) is required as a transcriptional cofactor of *LFY*. The *ufo* mutant exhibited a similar phenotype to the *lfy* mutant of *Arabidopsis*. The *HmLFY* and *HmUFO* screened in this study also interacted with *HmAP1-2*, *HmPI-1*, and *HmAP3-2* (Figure 7).

As the only gene in the ABCDE model that does not belong to the MADS-box family, *AP2* has the specific AP2 domain that encodes the AP2/EREBP family of transcription factors. The AP2 domain consists of approximately 60–70 amino acids, is highly conserved, can form an amphipathic α-helix, which is associated with protein interactions, has a nuclear localization signal sequence, and functions as a transcription factor [34]. The transcription factors of the AP2/EREBP gene family can be divided into three categories depending on the number of AP2 structural domains they contain, with the AP2 subfamily containing two AP2 structural domains in the protein structure. The subfamily can be divided into two categories, AP2 and ANT, and almost all of its members are closely related to plant development, such as the determination of flower organ morphology and development, the control of inflorescence meristem formation, and the normal development of ovules and seeds [35]. In floral organ development, *AP2* has similar functions to *AP1*, which belong to the same class A functional gene. They jointly control the development of calyx and petals and regulate genes related to floral meristem initiation, and *AP1* acts downstream of *AP2* [35]. As indicated by the similar phenotypes of the single mutants and the more pronounced tendency of the double mutants to shift from floral primordia to inflorescence, *AP1* and *AP2* have similar regulatory effects on floral primordia characteristics, with some superimposed effects, and together they influence the construction of floral organs [34]. Similarly, as an attribute gene of the floral meristem, *AP2* has a degree of mutual positive regulation with *LFY* gene expression (Figure 7) [36,37]. Molecular and genetic studies in *Arabidopsis thaliana* have shown that *AP2* was expressed at low levels in the stem apex, with an enhanced expression in the inflorescence meristem, and throughout the entire *Arabidopsis* floral development, i.e., immature buds, four whorls of floral organ primordia, and developed ovules and seeds [38]. In this study, a total of five *AP2* genes were screened in *H. macrophylla*, of which *HmAP2-1* and *HmAP2-2* had similar change trends, both reaching peak expression at late floral organ development, while *HmAP2-3*, *HmAP2-4*, and *HmAP2-5* demonstrated similar expression patterns, all expressed at high levels during the BS1 or BS2 stages and decreasing at later stages (Figure 8b). Although the RNA of *AP2* was detectable in both floral organs in whorls 3 and 4, it did not suppress *AG* expression in whorls 3 and 4. Chen et al. [39] suggested that this is because in whorls 3 and 4, *AP2*, a microRNA-mediated target site for gene regulation, is repressed at the translational level by miRNA172 and belongs to post-transcriptional silencing. Therefore, it is speculated that *HmAP2-1* and *HmAP2-2* may be responsible for regulating the normal development of ovules and seeds at a later stage and are sequence complementary to miRNA172 in whorls 3 and 4 and are repressed at the translational level, while *HmAP2-3*, *HmAP2-4,* and *HmAP2-5* may be responsible for the formation of floral meristem and the differentiation of sepals and petals and repress the expression of *AG* in whorl 1 and 2 at the early stage of floral organ formation. In addition, another member of the AP2 subfamily, *ANT*, has a partial functional overlap with *AP2* and is an important transcription factor that regulates ovule and female gamete development and can work in concert with *AP2* to repress *AG* expression in whorls 1 and 2 [34,40,41]. The *AIL5* gene within this group is also associated with flower development [35]. The protein–protein interaction network (Figure 7) illustrated that *AP2* interacted with the *ANT*, *AIL5*, *SVP,* and *AP3* genes related to flower development.

Class E genes are cofactors in flower development, which regulate the development of floral organs together with other classes of transcription factors, and play a role in the development of all floral organs in sepals, petals, stamens, carpels, and ovules [3,28]. In a study by Tsai et al. [42], it was suggested that class E and class A MADS-box genes were grouped together in the *AP1/AGL9 (AGAMOUS-like 9)* clade and the two were closely related. This is the same as the results of the phylogenetic analysis in this study (Figure 8a). At present, four *SEPALLATA (SEP)* genes have been identified in *Arabidopsis thaliana*, namely *SEP1 (AGL2)*, *SEP2 (AGL4)*, *SEP3 (AGL9),* and *SEP4 (AGL3)*. Neither single nor double mutants of the *SEP* gene differed much in the developmental phenotype [43]. In contrast, in the triple mutant of *sep1/2/3*, petals, stamens, and carpels were transformed into sepal-like floral organs, similar to the phenotype of the double mutant of the B- and C-class genes [43,44,45]; in the quadruple mutant of *sep1/2/3/4*, all floral organs were transformed into spiral leaf-like structures, consistent with the phenotype of the ABC-class triple mutant [24]. However, in the *sep* mutant, the expression of the class A, B, and C floral homeotic genes could be unaffected. These studies further reveal that class E genes are necessary to standardize the determinacy of all floral organs and floral meristems and that class A, B, and C genes play a key role in regulating floral organogenesis and development only when *SEP-like* gene expression is present in plants [32]. It is both a new floral organ determinant gene and an activator of ABC-like genes [32]. Furthermore, *SEP* genes are partially redundant in their function in controlling the identity of all floral organs, with *SEP4* determining the identity of sepals. In this study, three class E genes, *HmSEP1*, *HmSEP3-1,* and *HmSEP3-2*, were identified, but the *SEP4* gene, which regulates sepal development more obviously, was not screened. It is speculated that this gene presumably is not a differentially expressed gene and is not included in the screening. Alternatively, it may be that the deletion of this gene did not affect sepal formation due to factors such as functional redundancy. Alternatively, it is also possible that a change in gene function occurred during the evolutionary process of *H. macrophylla*, resulting in the development of sepals that do not require the *SEP4* gene or that the gene is expressed in the form of another gene. This also indicates that the regulatory network of floral organ development is complex and that the ABCDE model is universal in flowering plants while having numerous specificities.

**Figure 10:**
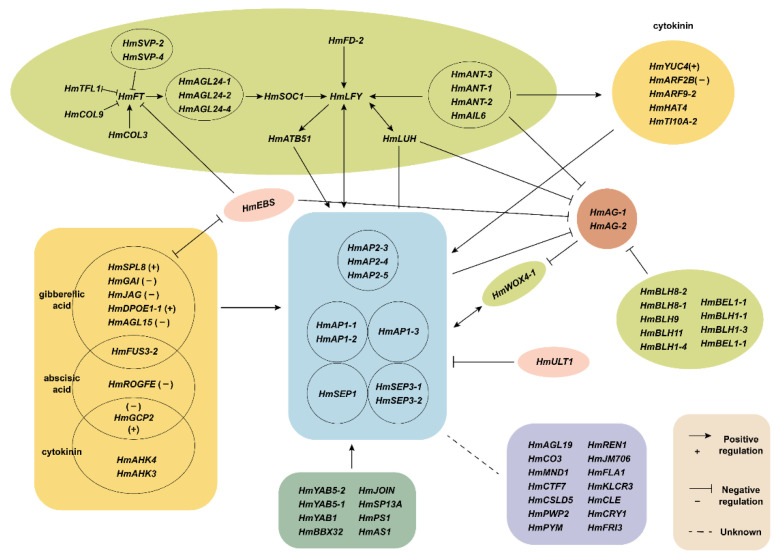
The hypothetical model of gene regulatory network for the development of decorative floret sepals in *H. macrophylla*.

In summary, this study postulated a gene regulatory network model for the development of decorative floret sepals in *H. macrophylla* (Figure 10). First, the core transcription factors controlling the development of decorative floret sepals, namely *HmAP1-2*, *HmAP1-1*, *HmAP1-3*, *HmAP2-3*, *HmAP2-4*, *HmAP2-5*, *HmSEP1*, *HmSEP3-1,* and *HmSEP3-2*, were identified based on gene expression patterns, phylogenetic analysis, and the ABCDE model. Subsequently, according to the protein–protein interaction network and literature studies, the genes *HmFT*, *HmSOC1*, *HmLFY,* and *HmLUH* were hypothesized to positively regulate class A and E transcription factors, and the *HmLFY*, *HmLUH,* and *ANT* genes also synergistically repressed *AG* gene expression in whorls 1 and 2. In addition, there were a number of genes associated with plant hormones, such as gibberellins, abscisic acid, cytokinins, and auxins, which were also involved in the sepal formation process at the early stages of flower development. It was hypothesized that they affected the development of decorative floret sepals by positively or negatively regulating the hormones. However, there were some genes with unknown functions, which also joined the developmental process of decorative florets, but their regulatory relationships on the process were not clear and needed further study. Although the present study hypothesized the functions and interrelationships of genes related to the development of decorative floret sepals in *H. macrophylla*, the conclusion is only a speculation. Its regulatory system has not been fully elucidated, and more in-depth experimental exploration is still needed.

## 4. Materials and Methods

### 4.1. Plant Materials

Perennial *H. macrophylla* ‘Endless Summer’ planted on the campus of Beijing Forestry University was selected as the test material. We selected plants with comparable growth to collect the buds and decorative flowers from the leaf bud state (BS1) to the full bloom stage (BS5), which were full of external morphology, healthy, and free from diseases and pests (Figure 2). Part of the fresh samples was used for morphological observation, and part was placed in centrifuge tubes and snap-frozen in liquid nitrogen tanks and stored in −80 °C ultralow temperature refrigerator for standby.

### 4.2. Transcriptome Sequencing

#### 4.2.1. Extraction and Detection of Total RNA from Transcriptome Sequencing Samples

Total RNA was extracted from samples at different developmental stages using the Aidlab EASYspin Plus RNA Extraction Kit (Aidlab Biotech, Beijing, China). The extracted RNA was tested for integrity, concentration, and purity by agarose gel electrophoresis combined with an ultramicro UV spectrophotometer (Thermo Fisher Scientific, Sunnyvale, CA, USA), and the samples that qualified were used for subsequent RNA high-throughput sequencing library construction. The sequencing unit was Shanghai OEBiotechnology Co., Ltd (Shanghai, China).

#### 4.2.2. Construction of cDNA Library and Transcriptome Sequencing

The mRNA was enriched from the total RNA by magnetic bead method and then broken into short fragments by adding interruption reagent, and the first strand of cDNA was synthesized by using short fragments of mRNA as the template with random primers. After synthesis of the second strand by DNA polymerase I, RNase H, dNTP, and buffer, the double-stranded cDNA was purified and end-repaired; poly A was added, and sequencing connectors were ligated. The library was prepared by PCR amplification after fragment size screening. The constructed libraries were quality-checked with an Agilent 2100 Bioanalyzer (Agilent Technologies, Santa Clara, CA, USA) and then paired-end sequenced using an Illumina sequencer (Genedenovo Biotechnology, Guangzhou, China).

#### 4.2.3. Sequencing Data Quality Monitoring and Sequence Assembly

The raw data obtained from high-throughput sequencing were filtered to remove the reads containing adapter, N ratio greater than 10%, all A bases, and low quality (the number of bases with Q ≤ 10 accounted for more than 50% of the whole read) to obtain clean reads. The transcripts were reconstructed and assembled by Trinity software (The Broad Institute, Cambridge, MA, USA) and CD-HIT software (http://www.bioinformatics.org/cd-hit/ (accessed on 4 March 2022)) to obtain the final unigenes for subsequent information analysis.

### 4.3. Bioinformatics Analysis and Screening of Differentially Expressed Genes

The obtained unigenes were annotated for gene function and aligned with the GO and KEGG databases to select the proteins and families with the highest sequence similarity. The sequence similarity alignment method was used to obtain the number of reads aligned to the unigene in each sample and calculate the expression abundance (FPKM value) of each unigene in each sample. Based on the magnitude of the FPKM value, the number of counts of each sample was standardized, and the difference fold was calculated and tested for significance. Eventually, the DEGs were screened with |log2Fold Change| ≥ 2 and the *p*-value ≤ 0.05 as the threshold. Trend analysis and PPI analysis were performed on the screened DEGs using the company’s cloud platform program (https://cloud.oebiotech.cn/ (accessed on 4 March 2022)). The trend analysis constructed the corresponding change trends by time points and then transformed the gene expression data and calculated the similarity of the transformed data with the corresponding change trends. Meanwhile, the time points were randomly disrupted, and the trend analysis was reperformed to count the number of genes in each trend. After performing numerous random rearrangements, an expected number of genes could be obtained in each trend, and finally, the *p*-value of the trend was calculated using the hypergeometric distribution algorithm. PPI analysis is a blast comparison of unigenes with allied species in the STRING database to obtain the interaction relationship and combined score of differential unigenes by homologous alignment substitution. The protein (gene) interaction network took the obtained list of target proteins (genes) and the possible two-by-two interactions between all proteins (or corresponding genes) of the species as input files and visualized the interaction relationships between proteins (genes) with high connectivity in a set of proteins (genes) in the form of a ring network. The up- and downregulation were distinguished by different colors according to the information on expression level changes. Phylogenetic tree was drawn with MEGA11 software (https://www.megasoftware.net/ (accessed on 31 July 2022)).

### 4.4. RT-qPCR Verification

To verify the accuracy of the transcriptome data, we selected 13 DEGs that might be associated with decorative floret formation for real-time quantitative PCR analysis. RNA was reverse transcribed into cDNA using the HiScript Ⅲ RT SuperMix for qPCR (+gDNA wiper) (Vazyme Biotech, Nanjing, China), which was used as a template for RT-qPCR. Gene-specific primers were designed using Primer Premier 5.0 software (Premier Biosoft, San Francisco, CA, USA) (Supplementary Table S7), and the reference gene was *EF1-β* [46]. The reaction system was prepared according to the instructions of TB Green^®^ *Premix Ex Taq*^TM^ II (Takara, Shiga, Japan), and PCR amplification was performed. The total reaction system was 20 μL, TB Green *Premix Ex Taq* II of 10 μL, 4 μL each of forward and reverse primers, and 2 μL of cDNA template. The amplification procedure was as follows: predenaturation at 95 °C for 30 s, denaturation at 95 °C for 5 s, annealing at 60 °C for 30 s, extension at 72 °C for 30 s, 40 cycles, melting curve. Each reaction was repeated three times. The relative expression of the target genes was calculated using the 2^−ΔΔCT^ method [47].

## 5. Conclusions

In this study, the transcriptomes of floral organs from the nutritional stage (BS1) to the blooming stage (BS5) of *H. macrophylla* were sequenced and aligned with the KEGG and GO databases. The DEGs of each comparative group were mainly enriched in the GO terms related to flower development, such as the maintenance of inflorescence meristem identity, floral whorl development, and pollen exine formation. The 347 DEGs obtained by differential expression analysis and GO functional enrichment were subjected to a trend analysis and a PPI analysis, and 17 ABCDE model-related transcription factors were identified and subjected to phylogenetic analysis and expression pattern analysis. Ultimately, this study postulated a model for the gene regulatory network of decorative sepal development in *H. macrophylla*, which laid the foundation for the study of the molecular mechanism of sepal formation in decorative florets.

## Figures and Tables

**Table 1:** Quality statistics of sample sequencing data.

Sample	Raw Reads	Raw Bases	Clean Reads	Clean Bases	Valid Bases	Q30	GC
BS1	51.67M	7.75G	50.52M	7.36G	94.96%	88.66%	45.55%
BS2	47.87M	7.18G	46.82M	6.84G	95.22%	91.14%	45.52%
BS3	50.73M	7.61G	49.60M	7.25G	95.34%	90.85%	45.37%
BS4	49.45M	7.42G	48.24M	7.06G	95.14%	91.86%	45.60%
BS5	47.49M	7.12G	46.46M	6.79G	95.31%	91.69%	45.38%

## Data Availability

All data generated or analyzed during this study are available.

## Supplementary Materials

The following supporting information can be downloaded at: https://www.mdpi.com/article/10.3390/ijms232214112/s1.
