# Supplementary figures and images for: Exploring the Molecular Mechanism of Sepal Formation in the Decorative Flowers of Hydrangea macrophylla ′Endless Summer′ Based on the ABCDE Model
Source: Int J Mol Sci. 2022 Nov 15;23(22):14112. doi: 10.3390/ijms232214112 (PMC9694991; doi:10.3390/ijms232214112)

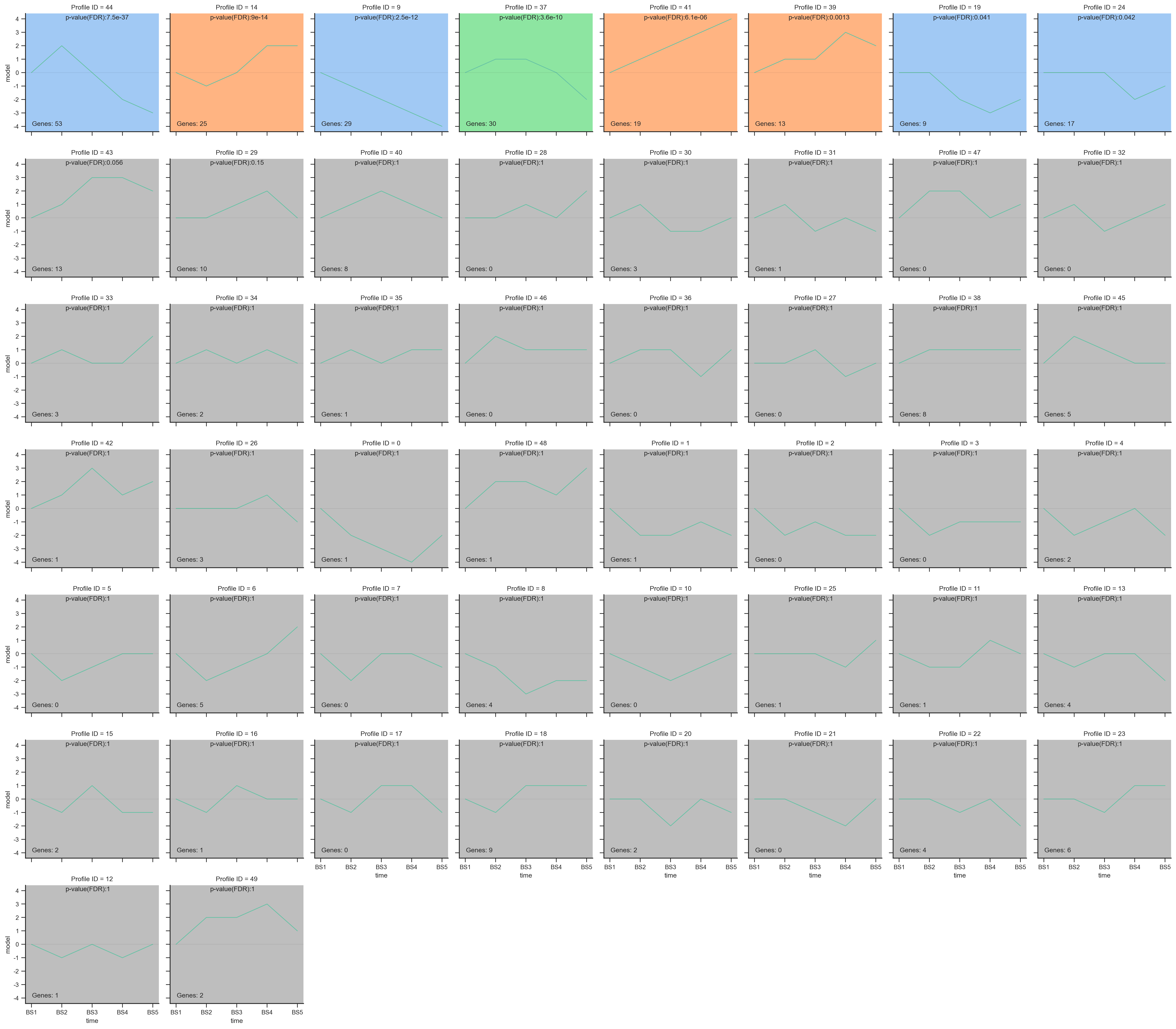

Supplement: Supplementary file 1 [file ijms-23-14112-s001.zip › Supplementary Figure S1.png]

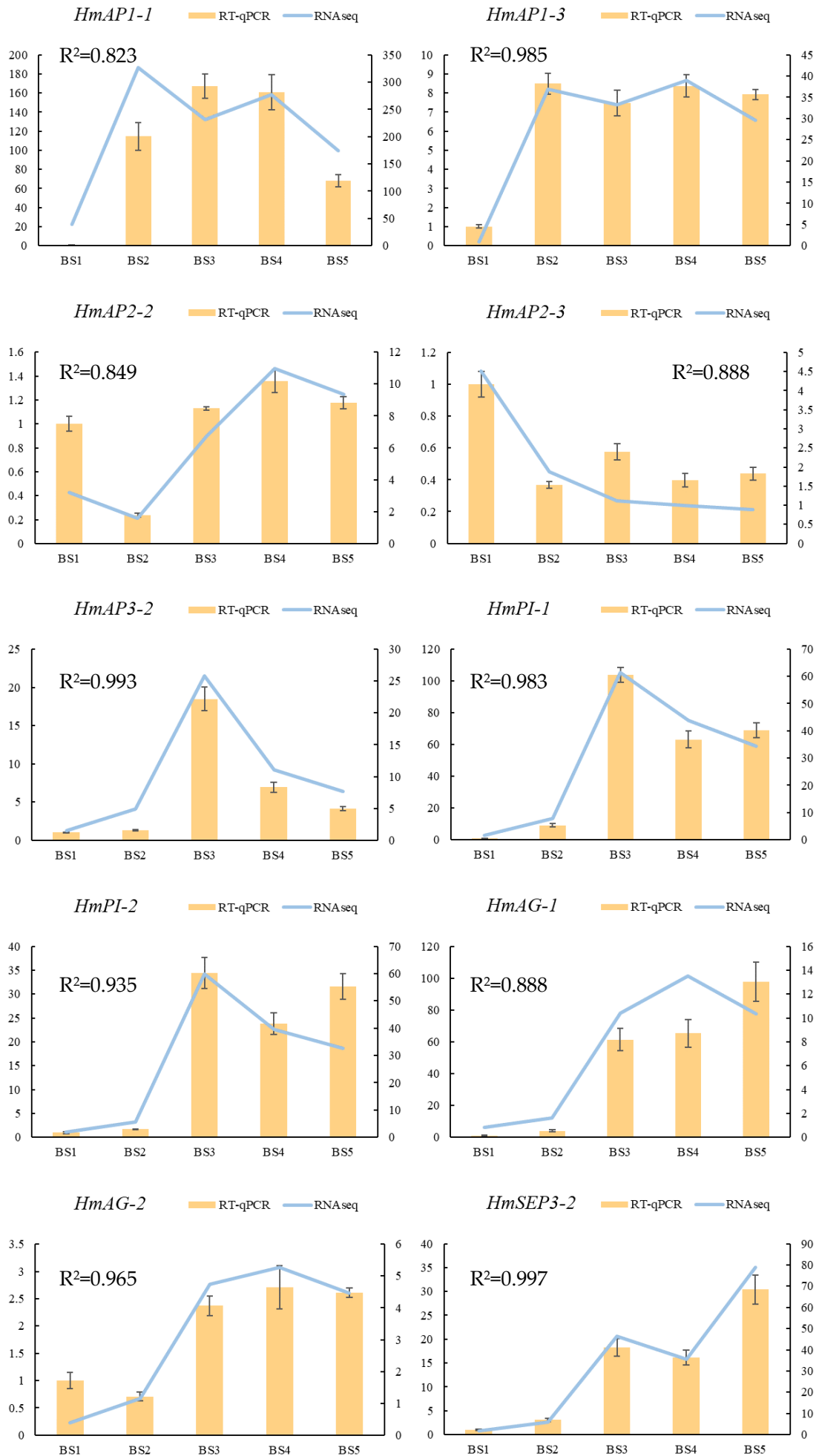

Supplement: Supplementary file 1 [file ijms-23-14112-s001.zip › Supplementary Figure S2.pdf]
